# Supplementary material for: Longevity gene responsible for robust blue organic materials employing thermally activated delayed fluorescence
Source: Nat Commun. 2023 Jul 3;14:3927. doi: 10.1038/s41467-023-39697-7 (PMC10318018; doi:10.1038/s41467-023-39697-7)
Supplement: Supplementary file 1 — Supplementary Information [file 41467_2023_39697_MOESM1_ESM.pdf]

Supplementary Information for Longevity Gene Responsible for Robust Blue Organic Emitters Employing Thermally Activated Delayed Fluorescence

Qing-Yu Meng,[1,‡] Rui Wang,[1,‡] Yi-Lei Wang,[2] Xing-Wei Guo,[2,3] Yu-Qi Liu,[1] Xue-Liang Wen,[1] Cheng-Yu Yao,[1] and Juan Qiao[1,4]✉

[1] Key Lab of Organic Optoelectronics and Molecular Engineering of Ministry of Education, Department of Chemistry, Tsinghua University, Beijing, China.

[2] Department of Chemistry, Tsinghua University, Beijing, China

[3] Center of Basic Molecular Science, Department of Chemistry, Tsinghua University, Beijing, China.

[4] Laboratory for Flexible Electronics Technology, Tsinghua University, Beijing, China

[‡] The authors contribute equally: Qing-Yu Meng, Rui Wang.

✉ E-mail: qjuan@mail.tsinghua.edu.cn.

Supplementary	Contents	Page
**Fig. 1**	The LDI-TOF-MS results (a) and proposed degradation mechanism (b) of DMAC-DPS, SpiroAC-Trz, DPAC-Trz, DCzTrz, DDCzTrz, and 5CzBN.	**S4**
**Fig. 2**	The PL spectrum of DMAC-DPS, DPAC-Trz, and DCzTrz in benzene.	**S4**
**Fig. 3**	Degradation tests of dilute and concentrated solution. a, PL spectrum of DMAC-DPS (5.0×10^{-5} M) in degassed and aerated solution. b, c, d, The degradation results of DMAC-DPS (a), DPAC-Trz (b) and DCzTrz (c) benzene solution under 380 nm.	**S5**
**Fig. 4**	Degradation tests of dilute and concentrated solution of DMAC-DPS in different light path length.	**S5**
**Fig. 5**	Degradation tests of TPPDA (a) and TBPe (b) benzene solution under 380 nm.	**S6**
**Supplementary**	**Discussions for photo-degradation tests in solution.**	**S6**
	Solvent Choice	**S6**
	Degassed dilute solution and aerated concentrated solution	**S6**
	Degradation tests of dilute and concentrated solution in different light path length.	
	Degradation tests of conventional fluorescence emitters	**S7**
**Fig. 6**	Transient PL decay curves of DMAC-DPS, SpiroAC-Trz, DPAC-Trz, DCzTrz, DDCzTrz, and 5CzBN. The black line is the decay curve of emitters and the red line is IRF.	**S8**
**Fig. 7**	Experimental and simulation results on neat films with assumption of $k_{nr,S}$=0 and $k_{nr,T}$=0.	**S9**
**Fig. 8**	Simulation results with/without TTA process under the assumption of $k_{nr,T}$ = 0 (a), and $k_{nr,S}$ = 0 (b).	**S10**
**Fig. 9**	The absorbance changes of the neat film of DMAC-DPS (a) and 5CzCN (b) before and after degradation.	**S10**
**Fig. 10**	Comparison of experimental (dots) and simulation (line) results of τ_p change in photo- degradation tests.	**S11**
**Fig. 11**	The phosphorescence spectrum of DMAC-DPS, SpiroAC-Trz, DPAC-Trz, DCzTrz, DDCzTrz, and 5CzBN at 77K and delayed 200 ms. (the onset energy for DMAC-DPS, DPAC-Trz, SpiroAC-Trz, 5CzTrz spectrum with no fine structure and the first peak energy for DCzTrz and DDCzTrz spectrum with fine structure).	**S11**
**Fig. 12**	Optimized structures of ADPS, SpiroAC-CN, DPAC-CN, and CzCN at S_0 and T_1 state.	**S11**
**Fig. 13**	IRC of C-X bond cleavage process at T_1 state of ADPS (a), SpiroAC-CN (b), DPAC-CN (c), and CzCN (d). The molecular structures in the figure correspond to the ends of the IRC curve and the energy maximum point.	**S12**
**Fig. 14**	The correlation between E_a and (BDE-E_{T1}) in a wide variety of TADF model molecules. The fragile bonds were labeled in red.	**S12**
**Fig. 15**	Chemical structures of 32 reported materials. The fragile bonds are labeled in red.	**S13**

**Fig. 16**	The correlation between BDE-E_{T1} and device lifetime of 32 reported materials (35 points).	**S14**
**Supplementary Discussions of materials exhibited exceptionally longer lifetime beyond the correlation.**		**S14**
**Fig. 17**	The correlation between BDE -E_{T1} and BDE -2E_{T1} of 32 reported materials in Supplementary fig. 15.	**S15**
**Fig. 18**	Correlation between device operational lifetime and k_{RISC} proposed by Lee et al. (a) and correlation between device operational lifetime and emission wavelength proposed by Zhang et al. (b)	**S16**
**Table 1**	Calculation equations for photophysical rate constants.	**S16**
**Table 2**	Molecular parameters of DMAC-DPS, SpiroAC-Trz, DPAC-Trz, DCzTrz, DDCzTrz, and 5CzBN in neat films.	**S17**
**Table 3**	Molecule parameters of ADPS, SpiroAC-CN, DPAC-CN, and CzCN at S_0 state and T_1 state (number in parentheses).	**S18**
**Table 4**	Molecule parameters, device lifetime, and host materials of some reported blue TADF emitters.	**S19**
**Fig. 19**	Chemical structures of host materials used in Supplementary Table 3.	**S19**
**Supplementary Discussions for numerical simulation.**		**S20**
	Jablonski diagram of the exciton dynamics in TADF materials by photo-excitation. (Supplementary Fig. 20)	**S20**
	Supplementary descriptions of exciton dynamics and kinetic equations (1)-(3).	**S20**
	Supplementary Discussions for the parameter choices in the numerical simulation.	**S20**
	Supplementary description for the simulation.	**S21**
	The output simulation results of DMAC-DPS neat film. (Supplementary Table 5)	**S21**
	Supplementary discussions for the rationality of assumptions in numerical simulation.	**S21**
	Supplementary discussions for the comparison between single exciton model and hot exciton models.	**S22**
**Supplementary References**		**S23**

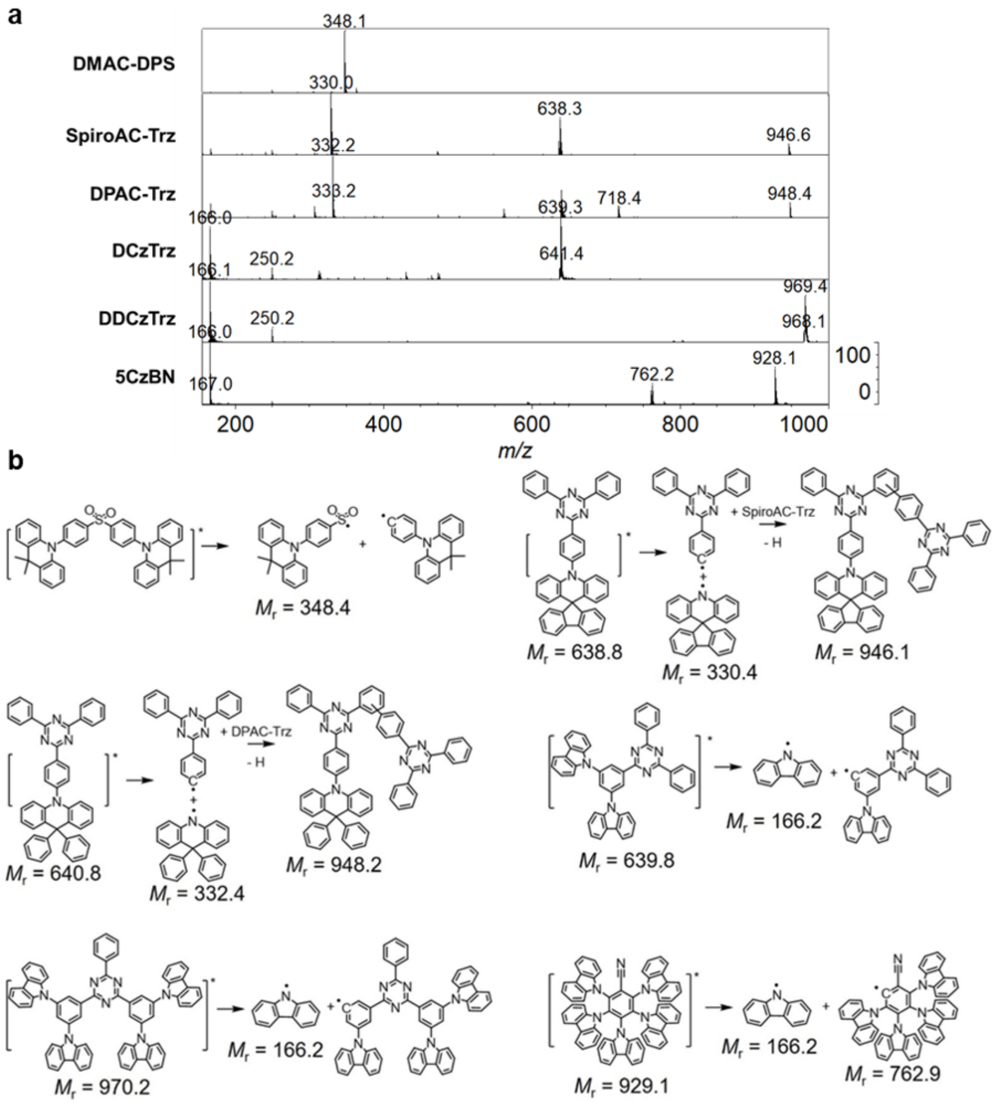

Supplementary Fig. 1 | The LDI-TOF-MS results (a) and proposed degradation mechanism (b) of DMAC-DPS, SpiroAC-Trz, DPAC-Trz, DCzTrz, DDCzTrz, and 5CzBN.

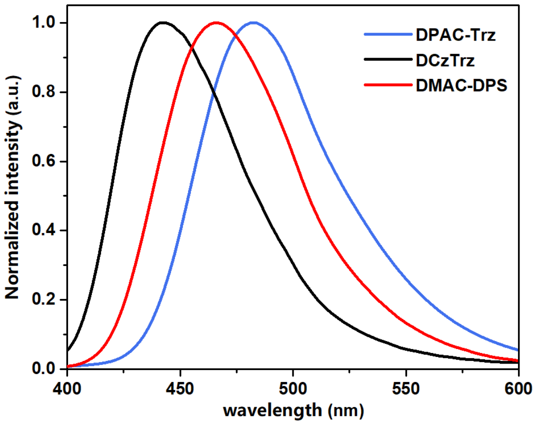

Supplementary Fig. 2 | The PL spectrum of DMAC-DPS, DPAC-Trz, and DCzTrz in benzene.

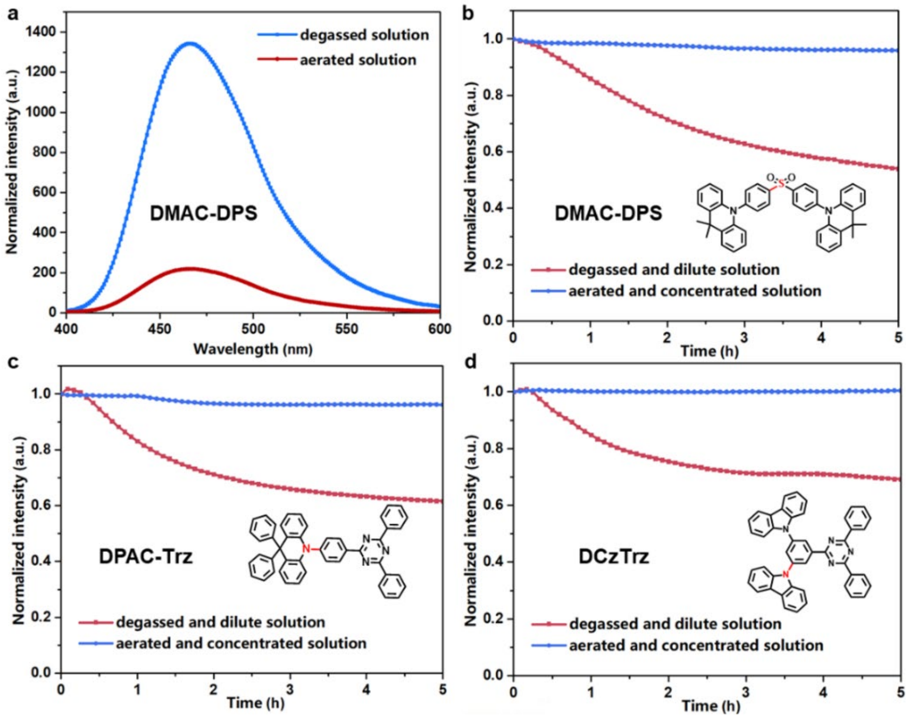

Supplementary Fig. 3 | Degradation tests of dilute and concentrated solution. a, PL spectrum of DMAC-DPS (5.0×10^{-5} M) in degassed and aerated solution. b, c, d, The degradation results of DMAC-DPS (a), DPAC-Trz (b) and DCzTrz (c) benzene solution under 380 nm.

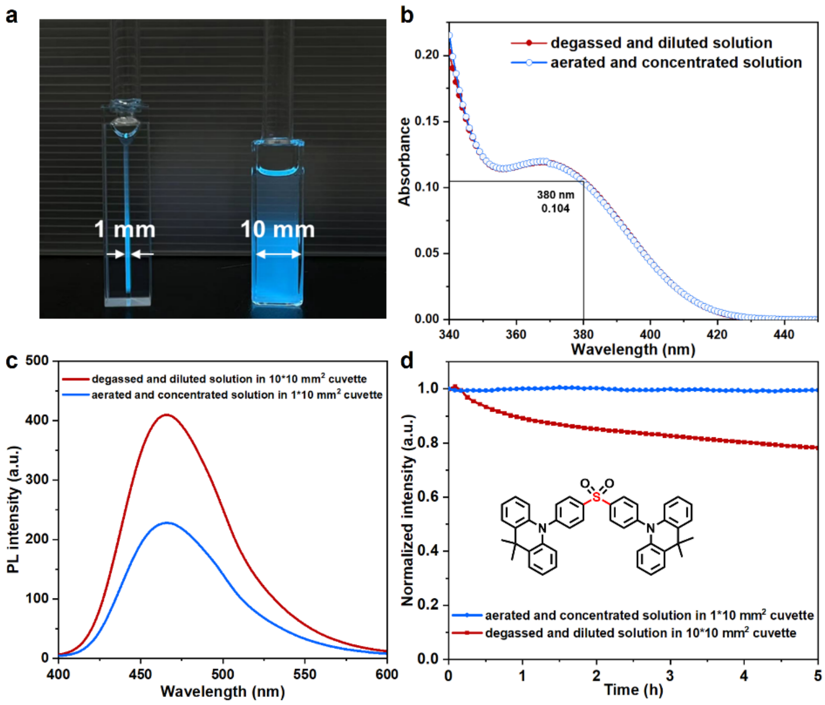

Supplementary Fig. 4 | Degradation tests of dilute and concentrated solution of DMAC-DPS in different light path length. a Cuvettes with light path of 1 mm (left) and 10 mm (right). b, c, d Absorption spectra (b), PL spectra (c), and UV degradation results under 380 nm illumination (d) of concentrated solution (5.0×10^{-4} M) in 1×10 mm^2 cuvette and diluted solution (5.0×10^{-5} M) in 10×10 mm^2 cuvette.

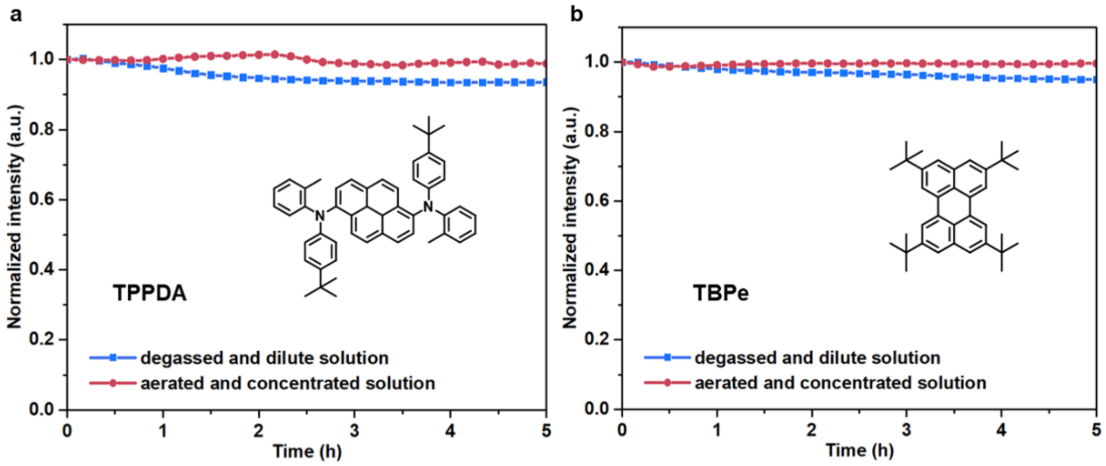

Supplementary Fig 5 | Degradation tests of TPPDA (a) and TBPe (b) benzene solution under 380 nm.

Supplementary Discussions for photo-degradation tests in solution.

Solvent Choice.

We choose benzene as the solvent as it is (1) highly stable and difficult to occur photo-induced reaction in the experimental condition, (2) no absorption at the excitation wavelength of 340-380 nm, (3) moderate polarity, and (4) good solubility.

Degassed dilute solution and aerated concentrated solution

To further support the conclusion that blue TADF materials mainly degrade at T_1 state, we conducted other 2 tests. We found that PL intensity of aerated solution is much weaker than that of degassed solution. For example, in DMAC-DPS, the PL intensity of aerated solution is only 16.3% of degassed solution (Supplementary Fig. 3a). This is because the PL of TADF emitters include prompt part and delayed part. The delayed part comes from RISC of T_1 excitons. Thus, once the T_1 excitons are quenched by O_2, the delayed PL will vanish, leading to the decreased PL intensity. On the other hand, the decreased PL intensity represents the decreased density of S_1 excitons, so based on the unchanged PL intensity of aerated solution and solution with 2-BP in photo-degradation tests, we could not directly exclude the effect of difference in density of S_1 excitons.

To solve this problem, we compared the degradation result of degassed dilute solution and aerated concentrated solution of DMAC-DPS, DPAC-Trz, and DCzTrz. The absorbance of concentrated solution is 0.993, much higher than that of dilute solution (0.167) in DMAC-DPS. Similar results occurred in DPAC-Trz and DCzTrz solution. The higher absorbance of concentrated solution makes up the decreased density of S_1 excitons in degassed solution since the initial density of S_1 excitons in concentrated solution is higher than that of dilute solution. As shown in Supplementary Figs. 3b-3d, in aerated and concentrated solution where the density of S_1 excitons is similar or even exceeds that in degassed and dilute solution, the PL intensity almost did not change since the T_1 excitons were quenched. In comparison, in the degassed dilute solution, the PL intensity shows visible decay. This comparison further confirmed that T_1 excitons have important influence on the degradation of TADF emitters rather than S_1 excitons.

Moreover, in the concentrated solution, the large gradation of exciton concentration might exist since the cuvette is 10×10 mm^2. To alleviate this undesirable effect, we further customized cuvettes with light path length of 1 mm (Supplementary Fig. 4a), and conducted the photo degradation tests of DMAC-DPS with concentrated solution in 1 mm cuvette and diluted solution in 10 mm cuvette with the same absorption of 0.104

(Supplementary Fig. 4b). As shown in Supplementary Fig. 4c, the initial PL intensity of aerated and concentrated solution with 1 mm light path is ~1/2 of that of diluted solution (228 and 415). Considering the fact that concentrated solution only has 1/10 emitting region of diluted solution. This PL intensity difference indicated that the density of S_1 exciton in the aerated and concentrated solution would be larger than that of in degassed and diluted solution. Meanwhile, in the 1×10 mm^2 cuvette, the gradation of exciton concentration would be alleviated. Thus, the degradation results in Supplementary Fig. 4d that the PL intensity of concentrated solution almost did not change since the T_1 excitons were quenched while the PL intensity of degassed dilute solution shows visible decay, strongly confirmed that TADF materials mainly degrade at T_1 state once again.

Degradation tests of conventional fluorescence emitters

To confirm our conclusion from another aspect, we conducted the degradation tests of conventional fluorescence emitters, namely, TPPDA and TBPe. The E_{S1} values of the two emitters are 2.82 and 2.80 eV, respectively. The E_{T1} values are 1.74 eV and 1.51 eV, respectively (obtained from DFT calculation). The difference between TPPDA and TBPe is TPPDA has C-N fragile bond while TBPe does not. We supposed if the S_1 excitons have important influence, the PL intensity of TPPDA would decay obviously. However, as shown in Supplementary Fig. 5, the PL intensity change of the two emitters is both below 5% neither in the degassed solution nor in the solution.

In summary, based on the contrast experiments on concentrated and dilute solution and the degradation tests of conventional fluorescence emitters, we further confirmed the conclusion that blue TADF emitters mainly degrade at T_1 state rather than S_1 state, laying the solid groundwork for our further explorations.

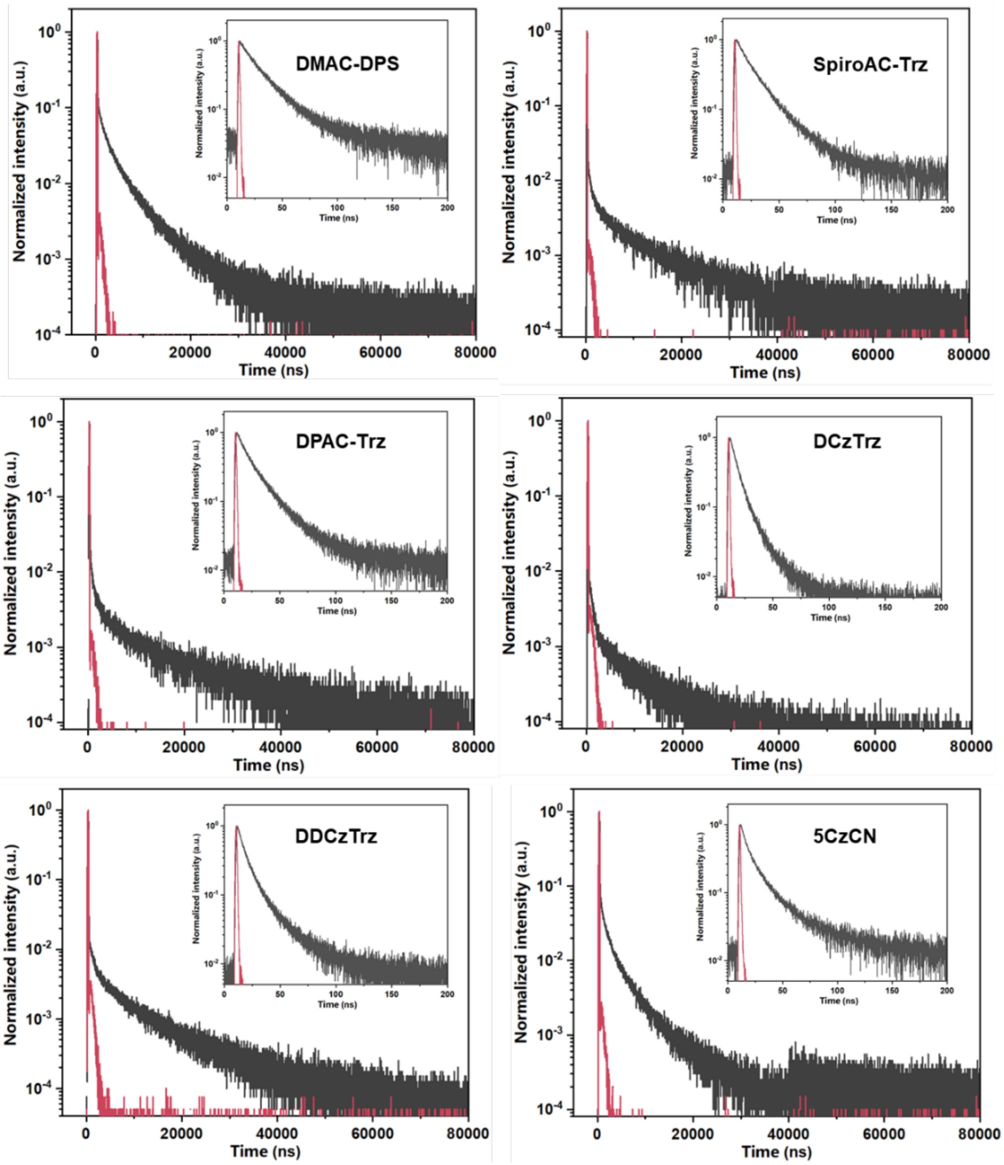

Supplementary Fig 6 | Transient PL decay curves of DMAC-DPS, SpiroAC-Trz, DPAC-Trz, DCzTrz, DDCzTrz, and 5CzBN. The black line is the decay curve of emitters and the red line is IRF.

Supplementary Note 1

In order to obtain the photophysical parameters required for the simulation, we measured the transmittance, PLQY (Φ), and the transient decay curve of the 80 nm pure films of each material (Supplementary Fig. 6). Then the corresponding values of I, k_{r}, k_{nr}, k_{ISC} and k_{RISC} are calculated by the following formulas in Supplementary Table 1[1].

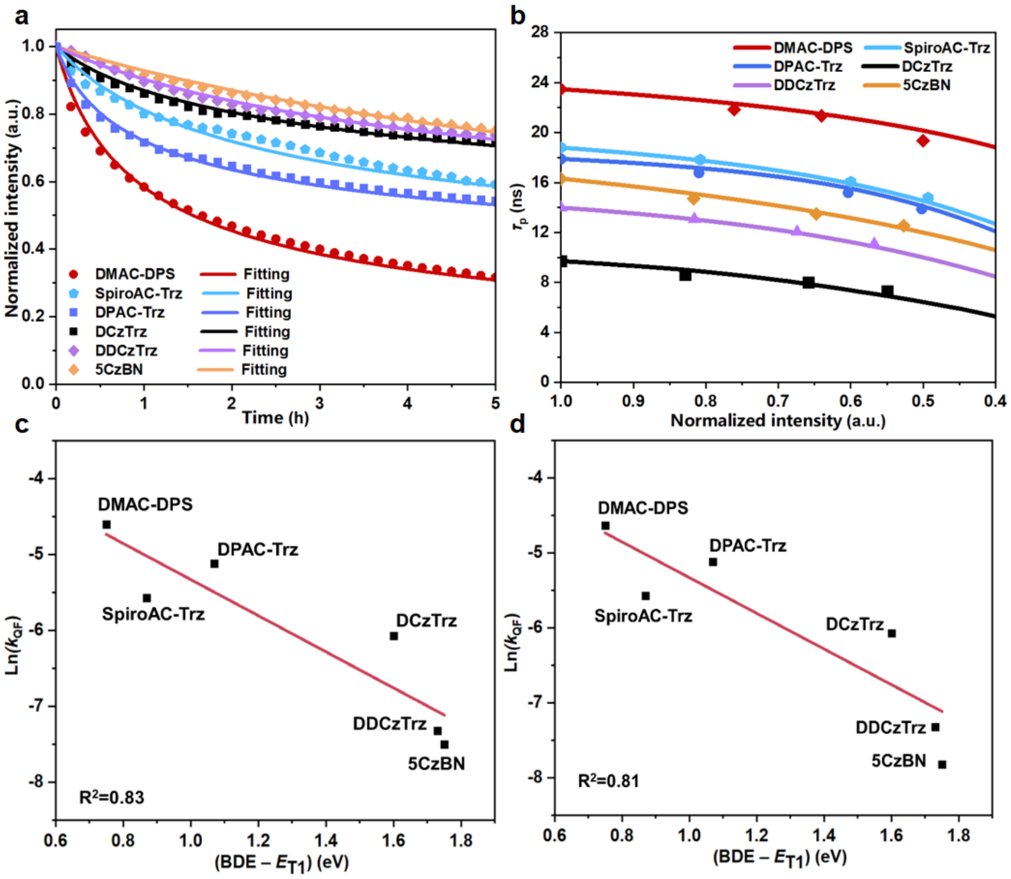

Supplementary Fig 7 | a Experimental and simulation results on neat films of DMAC-DPS, SpiroAC-Trz, DPAC-Trz, DCzTrz, DDCzTrz, and 5CzBN with the thickness of 80 nm. **b** Comparison of experimental (dots) and simulation (line) results of τ_p change in photo- degradation tests. **c** The correlation between the quencher formation rate (k_{QF}) and BDE-E_{T1}. All results were simulated with the assumption of $k_{nr,S}$=0. **d** The correlation between the quencher formation rate (k_{QF}) and BDE-E_{T1} with the assumption of $k_{nr,T}$=0.

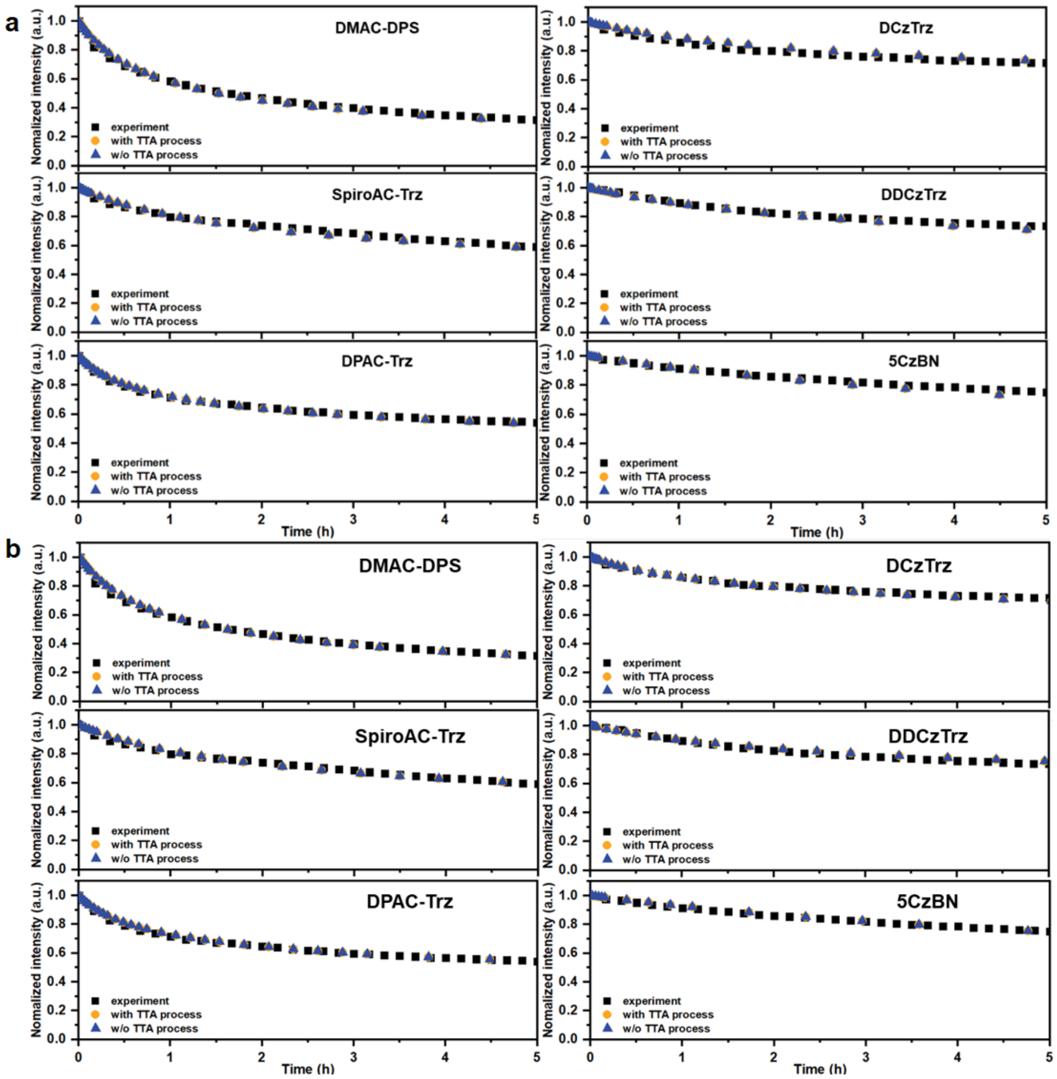

Supplementary Fig 8 | Simulation results of DMAC-DPS, SpiroAC-Trz, DPAC-Trz, DCzTrz, DDCzTrz, and 5CzBN with/without TTA process under the assumption of $k_{nr,\,T} = 0$ (a), and $k_{nr,\,S} = 0$ (b).

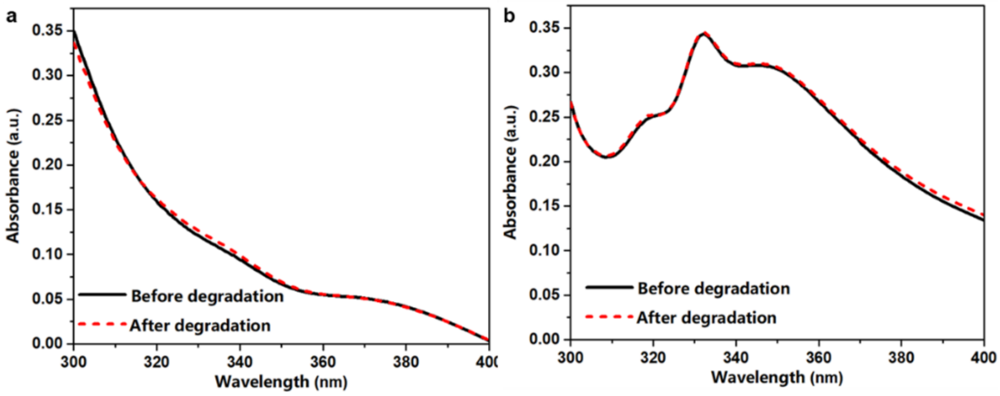

Supplementary Fig 9 | The absorbance changes of the neat film of DMAC-DPS (a) and 5CzCN (b) before and after degradation.

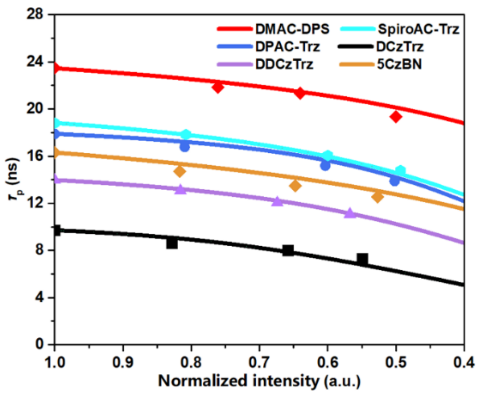

Supplementary Fig 10 | Comparison of experimental (dots) and simulation (line) results of τ_p change in photo- degradation tests.

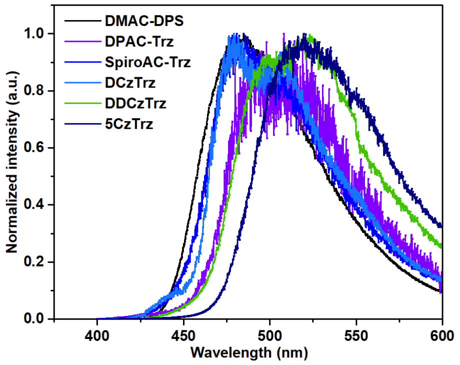

Supplementary Fig 11 | The phosphorescence spectrum of DMAC-DPS, SpiroAC-Trz, DPAC-Trz, DCzTrz, DDCzTrz, and 5CzBN at 77K and delayed 200 ms. (the onset energy for DMAC-DPS, DPAC-Trz, SpiroAC-Trz, 5CzTrz spectrum with no fine structure and the first peak energy for DCzTrz and DDCzTrz spectrum with fine structure).

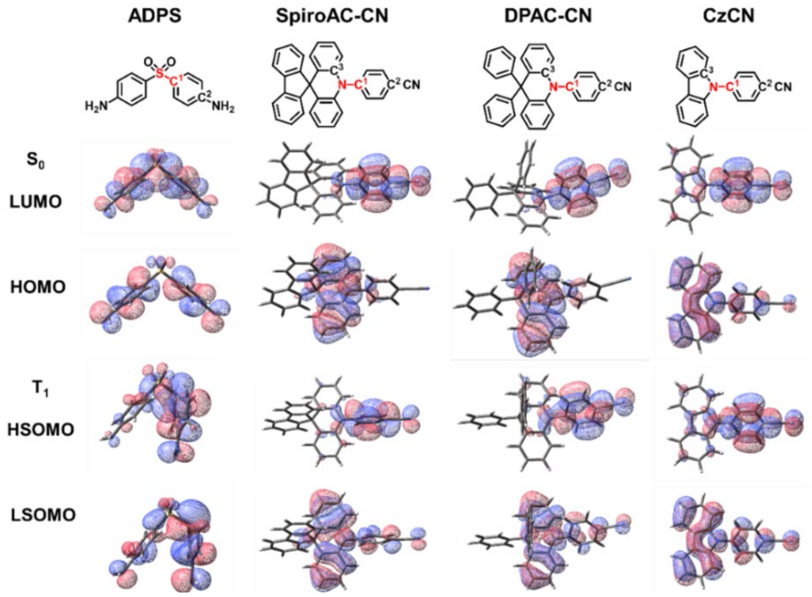

Supplementary Fig 12 | Optimized structures of ADPS, SpiroAC-CN, DPAC-CN, and CzCN at S_0 and T_1 state.

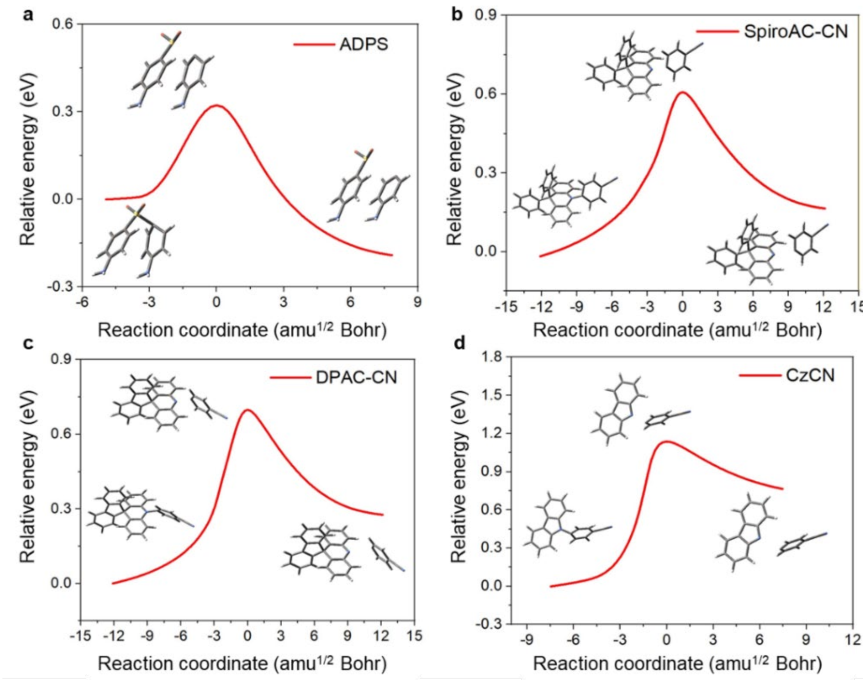

Supplementary Fig 13 | IRC of C-X bond cleavage process at T_1 state of ADPS (a), SpiroAC-CN (b), DPAC-CN (c), and CzCN (d). The molecular structures in the figure correspond to the ends of the IRC curve and the energy maximum point.

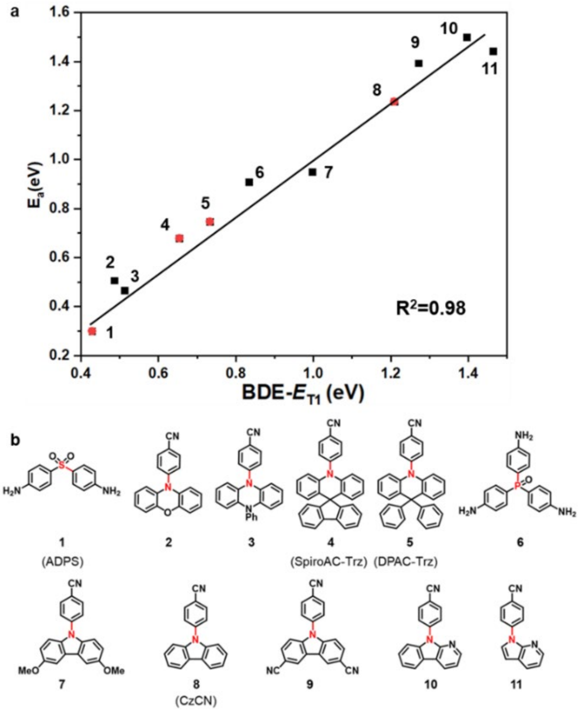

Supplementary Fig 14 | a The correlation between E_a and (BDE-E_{T1}) in a wide variety of TADF model molecules. b The chemical structures of the model molecules. The fragile bonds were labeled in red.

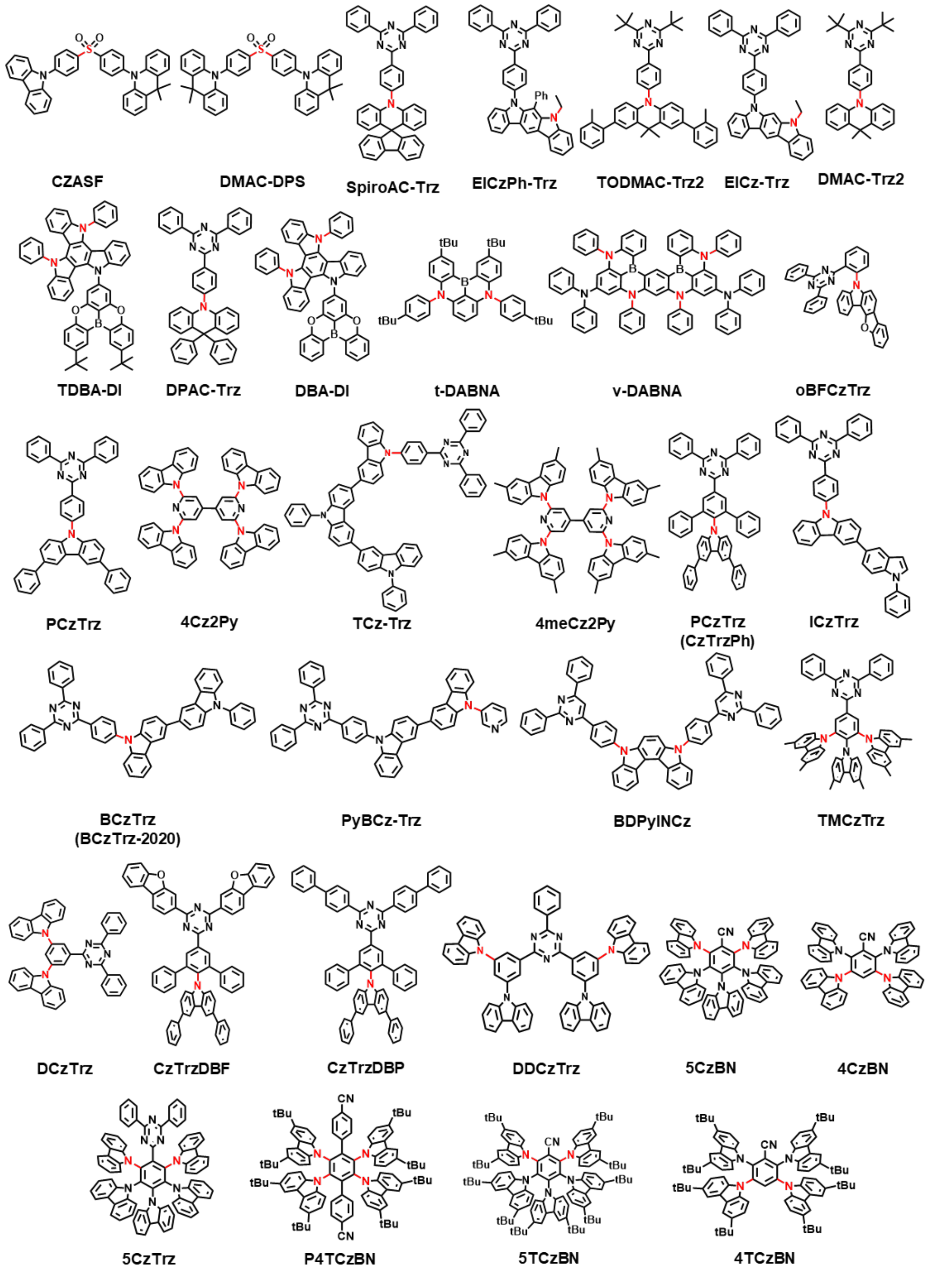

Supplementary Fig 15 | Chemical structures of 32 reported TADF materials with emission peak less than 500 nm. The fragile bonds are labeled in red.

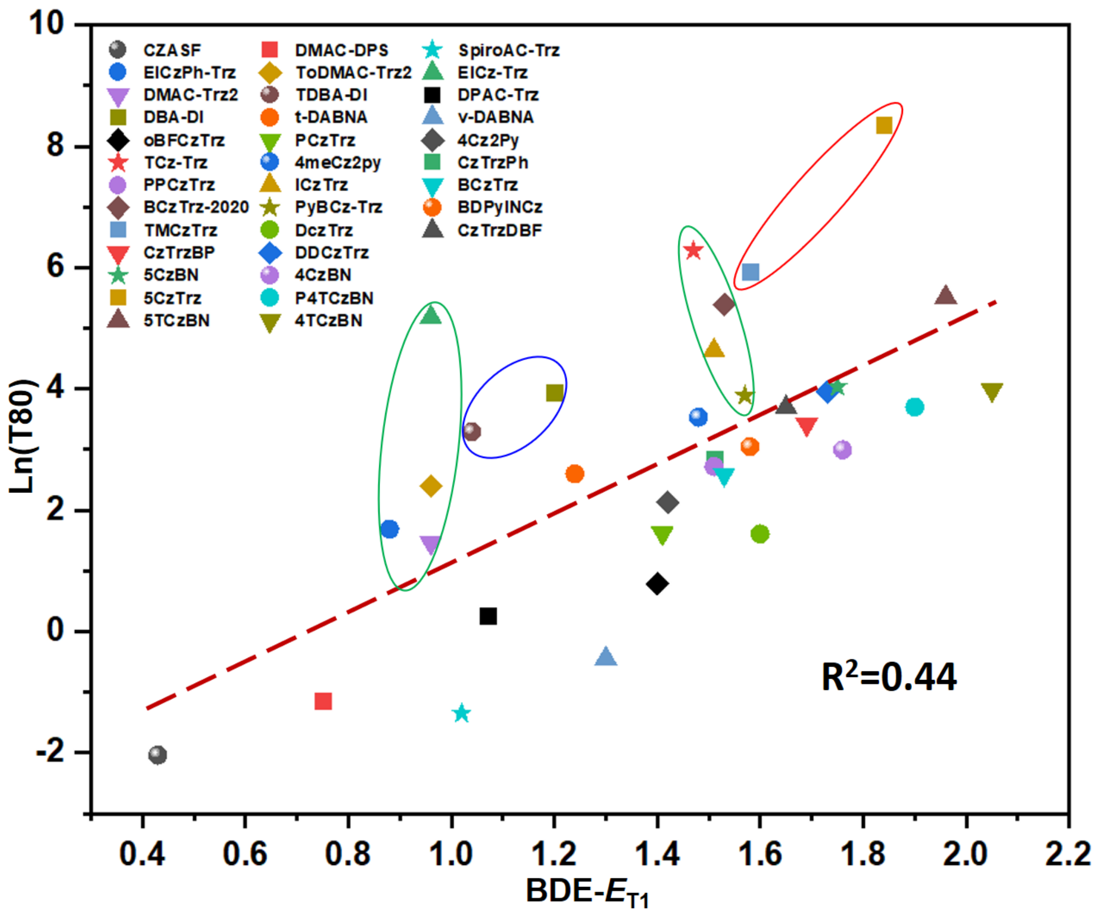

Supplementary Fig 16 | The correlation between BDE_f -E_{T1} and device lifetime of 32 reported materials (35 points).

Supplementary Discussions of materials exhibited exceptionally longer lifetime beyond the correlation.

Firstly, in principle, since RISC is a competition process for bond cleavage at T_1 state, the kinetic parameter k_{RISC} would also be important for improving molecule intrinsic stability or device lifetime. Indeed, Lee et al. demonstrated a good linearity correlation between k_{RISC} of TADF emitters and corresponding device operational lifetime[2]. Yet, this correlation was obtained from 4 TADF emitters featuring very similar molecule structures. To explore its generality, we tried to collect k_{RISC} values of the above 35 points from literatures, but got only that of 13 points because the others' were not reported. Unfortunately, we didn't find likewise good linearity correlation in such a wide variety of materials (Supplementary Fig 18a). It might originate from the high sensitivity of k_{RISC} value to different measurement and calculation methods. This result demonstrated again that as for the "trait" of device lifetime, thermodynamic parameter BDE-E_{T1} can act as the intrinsic molecule "longevity gene", which is particularly valuable for high throughput virtual screening and material design. During "gene expression", "environment" such as carrier balance or host-guest interactions, would also have important influence on the "trait" of device lifetime because they could greatly affect the kinetic molecule parameters such as k_{RISC}. For DBA-DI, TDBA-DI , and 5CzTrz, they all possess high k_{RISC} values (6.21×10^6 s^{-1}, 1.08×10^6 s^{-1}, and 1.5×10^7 s^{-1}, respectively). Therefore, the longer operational lifetime of these 3 materials could also be partially attributed to the superior k_{RISC}.

Secondly, 9 points reported by Zhang et.al (in the green circle) also did not fit the correlation well[3]. These materials have very comparable BDE values (~4.2 eV for carbazole derivatives and ~3.6 eV for DMAC

derivatives) and E_{T1} values (~2.7 eV), which leads to very comparable BDE-E_{T1} values (~1.5 eV for carbazole derivatives and ~0.9 eV for DMAC derivatives). Therefore, in the devices based on these materials, the difference in device lifetime is no longer decided by BDE-E_{T1}, but other factors because they were evaluated in the same device structure. Indeed, in the original literature Zhang et. al explained the different device lifetime by different k_r and EL emission wavelength of those carbazole derivatives in different host materials.

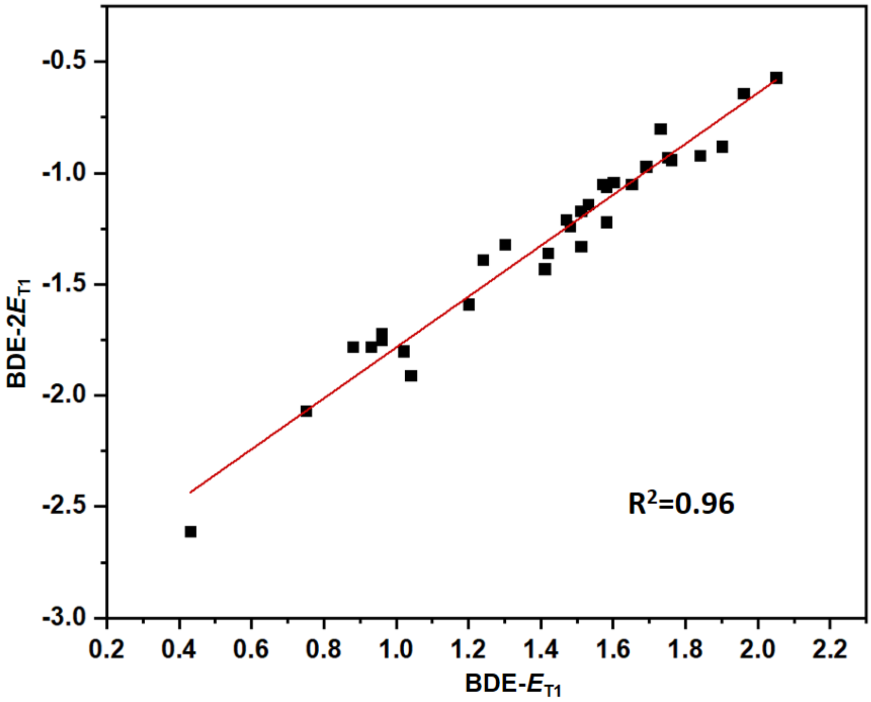

Supplementary Fig 17 | The correlation between BDE -E_{T1} and BDE -2E_{T1} of 32 reported materials in Supplementary Fig. 15.

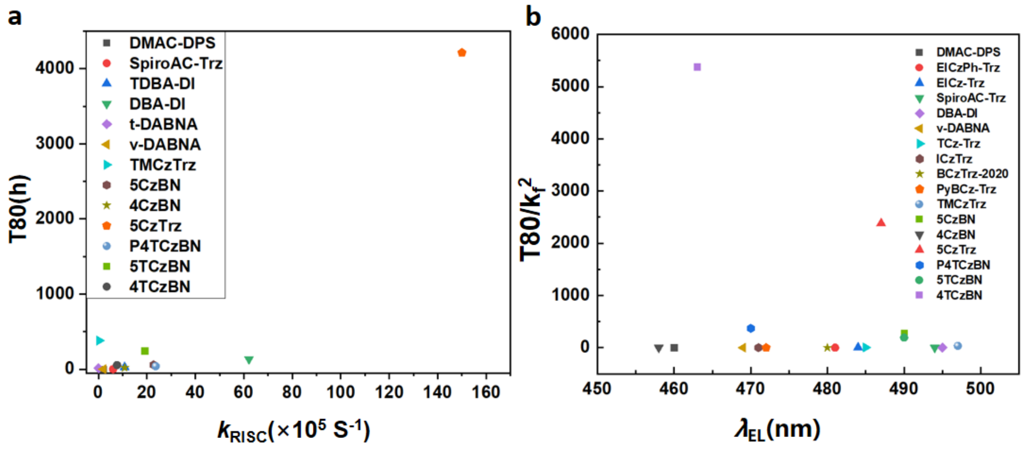

Supplementary Fig 18 | a Correlation between device operational lifetime and k_{RISC} proposed by Lee et al. b Correlation between device operational lifetime and emission wavelength proposed by Zhang et al.

Supplementary Table 1. Calculation equations for photophysical rate constants.

$I = \frac{P\lambda\cos\theta(1-T)}{\mathrm{h}cd}$	
$\Phi_p = \frac{k_r}{k_p} = \frac{(A_p+A_d)k_d}{A_pk_d+A_dk_p}\Phi_{PLQY}$	
$\Phi_d = \Phi_p\sum_{i=1}^{\infty}(\Phi_{ISC}\Phi_{RISC})^i = \frac{(k_p-k_d)A_d}{A_pk_d+A_dk_p}\Phi_{PLQY}$	
$k^S = k_p - k_d\frac{\Phi_d}{\Phi_p} + k_{ISC}\frac{\Phi_{r,T}}{\Phi_{r,S}}$	
With the assumption of $k_{nr,T} = 0$, $k_{r,T}=0$	With the assumption of $k_{nr,S} = 0$, $k_{r,T}=0$, $k^S \sim k_p$
$k_p = \tau_p^{-1} = k_r + k_{ISC}(1+\frac{k_{RISC}}{k^S-k_d}) + k_{nr,S}$	$k_p = \tau_p^{-1} = k_r + k_{ISC}$
$k_d = \tau_d^{-1} = k_{RISC}(1-\frac{k_{ISC}}{k^S-k_d})$	$k_d = \tau_d^{-1} = k_{nr,T} + k_{RISC}(1-\frac{k_{ISC}}{k_p})$
$k_{ISC} = k_p\frac{\Phi_d}{\Phi_{PLQY}} + k_d\frac{\Phi_d}{\Phi_p}$	$k_{ISC} = k_p(1-\Phi_p)$
$k_{RISC} = k_d\frac{\Phi_{PLQY}}{\Phi_p}$	$k_{RISC} = \frac{k_pk_d}{k_{ISC}}\frac{\Phi_d}{\Phi_p}$

Supplementary Note 2

In the above equations, P is the excitation power density of 1.48 and 2.10 mW cm^{-2} at 340 nm and 380 nm, respectively. λ is the excitation wavelength, and the λ corresponding to DMAC-DPS, DCzTrz, DDCzTrz and 5CzBN films is 340 nm. Since the absorption of DPAC-Trz and SpiroAC-Trz films is too high at 340 nm, the λ used is 380 nm. θ is the angle between excitation light and normal of film (30°); h is Plank constant; c is the speed of light; d is film thickness; k_p and τ_p are the consumption rate constant and lifetime of the prompt excitons after excitation, respectively. k_d and τ_d are the consumption rate constant and lifetime of the delayed excitons, respectively. Φ_{ISC} and Φ_{RISC} are the quantum yields of ISC and RISC processes, respectively. Φ_p and Φ_d are the quantum yields of prompt and delayed emission. k^S is the singlet exciton decay rate. A_p and A_d are the pre-exponential factor of fitting curve for prompt and delayed components.

Supplementary Table 2. Molecular parameters of DMAC-DPS, SpiroAC-Trz, DPAC-Trz, DCzTrz, DDCzTrz, and 5CzBN in neat films.

Material	DMAC-DPS	SpiroAC-Trz	DPAC-Trz	DCzTrz	DDCzTrz	5CzBN
T(Transmissivity)	0.80	0.88	0.84	0.66	0.58	0.49
Φ/Φ_d	0.77/0.62	0.87/0.43	0.72/0.33	0.14/0.034	0.22/0.089	0.52/0.37
τ_p (ns)	23.5	18.8	17.9	9.7	14.0	16.3
τ_d (μs)	4.4	10.4	8.9	6.6	9.2	3.6
I (10^{20} s^{-1})	0.55	0.50	0.67	0.91	1.1	1.4
k_r (10^7 s^{-1})	0.60	2.33	2.17	1.12	0.96	0.96
k_{ISC} (10^7 s^{-1})	3.66 (3.57)	2.99 (2.65)	3.42 (2.58)	9.20 (2.48)	6.19 (2.85)	5.17 (4.36)
$k_{nr,S}$ (10^6 s^{-1})	0 (0.85)	0 (3.40)	0 (8.47)	0 (67.1)	0 (33.4)	0 (8.12)
k_{RISC} (10^5 s^{-1})	11.6 (12.2)	1.68 (1.91)	1.56 (2.08)	0.54 (2.00)	0.82 (1.79)	7.61 (8.76)
$k_{nr,T}$ (10^4 s^{-1})	6.17 (0)	2.23 (0)	5.20 (0)	14.6 (0)	9.72 (0)	15.5 (0)
k_{QS} (10^{-9} cm^3 s^{-1})	0.93 (0.74)	1.1 (1.0)	0.96 (1.05)	3.4 (6.3)	1.7 (2.7)	3.1 (2.5)
k_{QT} (10^{-11} cm^3 s^{-1})	0.39 (0.30)	1.0 (0.9)	2.1 (2.1)	3.8 (6.1)	1.7 (1.8)	0.54 (0.30)
k_{QF} (10^{-3} s^{-1})	9.6 (9.7)	2.8 (3.8)	4.6 (6.0)	1.3 (2.3)	0.42(0.66)	0.29 (0.40)
BDE (eV)	3.57	3.69	3.83	4.24	4.26	4.43
E_{T1} (eV)	2.82	2.76	2.69	2.64	2.53	2.64
BDE- E_{T1} (eV)	0.75	0.93	1.14	1.60	1.73	1.79

Supplementary Note 3

1. Values of $k_{r,S}$, k_{ISC}, k_{RISC}, and $k_{nr,T}$ ($k_{nr,S}$) were obtained from experiments based on the method proposed by Adachi et al.[1] with the assumption of $k_{nr,S} = 0$ and $k_{nr,T} = 0$ (values in parentheses), respectively.

2. k_{QF} values were obtained though numerical simulations.

3. BDE values are calculated at M06-2X-D3 6-31G* level.

4. E_{T1} values were obtained from the PL spectrum (Supplementary Fig. 11) (the onset energy for spectrum with no fine structure and the first peak energy for spectrum with fine structure).

Supplementary Table 3. Molecule parameters of ADPS, SpiroAC-CN, DPAC-CN, and CzCN at S_0 state and T_1 state (number in parentheses).

Material	ADPS	SpiroAC-CN	DPAC-CN	CzCN
Bond length of C−X bond (Å)	1.777 (1.798)	1.426 (1.395)	1.423 (1.392)	1.408 (1.382)
∠XC1C2 (°)	178.7 (125.1)	179.5 (177.0)	178.6 (176.1)	180.0 (180.0)
C3-NC1-C2 (°)	-	82.4 (54.7)	88.4 (60.0)	52.1 (45.0)

Supplementary Table 4. Molecule parameters, device lifetime, and host materials of the reported blue TADF emitters in literatures.

Material	E_{T1} (eV)	BDE_f (eV)	BDE_f- E_{T1} (eV)	T80@500 cd m^{-2} (h)	Ln(T80)	Host	Ref.
CzASF	3.04	3.47	0.43	0.13	-2.04		4
DMAC-DPS	2.82	3.57	0.75	0.32	-1.14	DPEPO	5
SpiroAC-Trz	2.82	3.69	0.87	0.26	-1.35	mCPCN	6
EICzPh-Trz	2.66	3.54	0.88	5.42	1.69	mCBP	3
ToDMAC-Trz2	2.71	3.64	0.93	11.00	2.40	PPT	7
EICz-Trz	2.68	3.64	0.96	177.61	5.18	mCBP	3
DMAC-Trz2	2.71	3.67	0.96	4.30	1.46	PPT	7
TDBA-DI	2.95	3.99	1.04	26.90	3.29	mCBPCN	8
DPAC-Trz	2.76	3.83	1.07	1.29	0.25	DPEPO	9
DBA-DI	2.79	3.99	1.20	134.40	4.90	mCBPCN	10
t-DABNA	2.63	3.87	1.24	13.45	2.60	mCBP	11
v-DABNA	2.62	3.92	1.30	0.64	-0.45	DOBNA-OAr	12
oBFCzTRZ	3.00	4.40	1.40	2.20	0.79	DPEPO	13
PCzTrz	2.84	4.25	1.41	5.05	1.62	CBP:CNmCBPCN	14
4Cz2Py	2.78	4.20	1.42	8.40	2.13	PPT	15
TCz-Trz	2.68	4.15	1.47	532.84	6.28	mCBP	3

4meCz2Py	2.72	4.20	1.48	34.20	3.53	PPT	15
PPCzTrz	2.84	4.35	1.51	15.14	2.72	CBP:CNmCBPCN	14
CzTrzPh	2.84	4.35	1.51	17.20	2.84	CNmCBPCN	16
ICz-Trz	2.68	4.19	1.51	102.89	4.63	mCBP	3
BCz-Trz-2020	2.67	4.20	1.53	219.80	5.39	mCBP	3
BCzTrz	2.67	4.20	1.53	13.33	2.59	SF3K	9
PyBCz-Trz	2.62	4.19	1.57	48.74	3.89	mCBP	3
BDPyINCz	2.64	4.22	1.58	21.00	3.04	mCBPCN	3
TMCzTrz	2.80	4.38	1.58	607.40	6.41	mCBP	17
DCzTrz	2.64	4.24	1.60	5.00	1.61	mCBP	18
CzTrzDBF	2.70	4.35	1.65	40.30	3.70	CNmCBPCN	16
CzTrzBP	2.66	4.35	1.69	30.50	3.42	CNmCBPCN	16
DDCzTRz	2.53	4.26	1.73	52.00	3.95	mCBP	18
5CzBN	2.68	4.43	1.75	56.70	4.04	mCBP	19
4CzBN	2.70	4.46	1.76	20.00	3.00	mCBP	19
5CzTrz	2.76	4.60	1.84	4216.00	8.35	mCBP	17
P4TCzBN	2.78	4.68	1.90	40.40	3.70	mCPCz	11
5TCzBN	2.60	4.56	1.96	247.90	5.51	mCBP	19
4TCzBN	2.62	4.67	2.05	53.80	3.99	mCBP	19

Supplementary Note 4

Material and data collection criteria:

1. EL emission peak<500 nm.
2. E_{T1} is obtained from the PL spectrum reported in the literature (the onset energy for spectrum with no fine structure and the first peak energy for spectrum with fine structure).
3. BDE values are calculated by M06-2X-D3/6-31G*.
4. T80@500 cd/m^2 is obtained from the literature or calculated by T_X/T_y (@ the same initial luminance) =Ln(x/100)/Ln(y/100) and $(X/Y)^n$=Constant, X, Y are the different initial luminance and n=1.75.

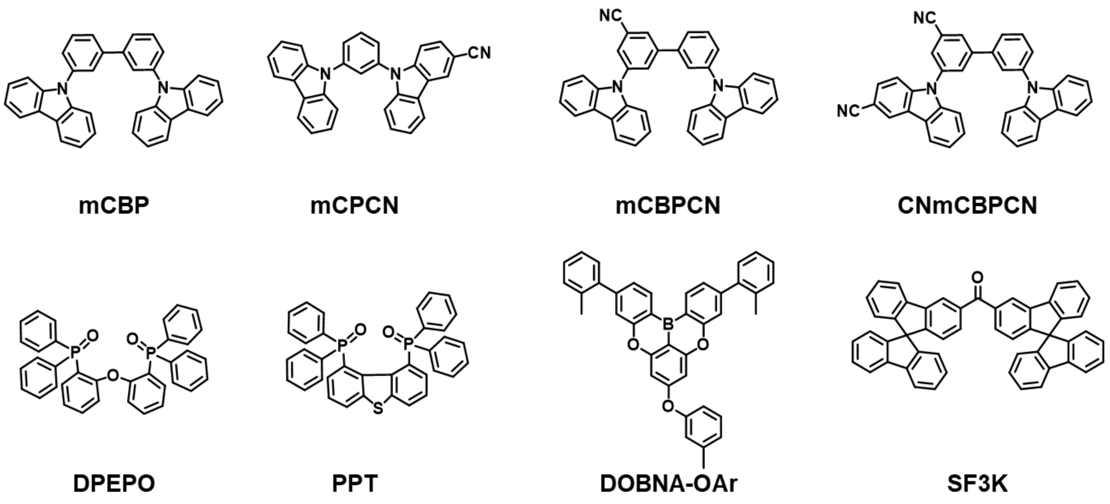

Supplementary Fig 19 | Chemical structures of host materials used in Supplementary Table 3.

Supplementary Discussions for numerical simulation.

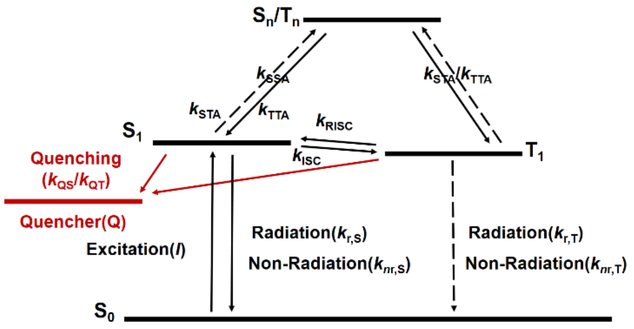

Supplementary Fig 20 | Jablonski diagram of the exciton dynamics in TADF materials by photo-excitation.

$$\frac{\mathrm{d}n_S}{\mathrm{dt}} = I - (k_{r,S} + k_{nr,S} + k_{ISC})n_S + k_{RISC}n_T - (k_{SSA}n_S^2 + k_{STA}n_S n_T - \gamma k_{TTA}n_T^2) - k_{QS}n_S n_Q \quad (1)$$

$$\frac{\mathrm{d}n_T}{\mathrm{dt}} = k_{ISC}n_S - (k_{r,T} + k_{nr,T} + k_{RISC})n_T - (1+\gamma)k_{TTA}n_T^2 - k_{QT}n_T n_Q - k_{QF}n_T \quad (2)$$

$$\frac{\mathrm{d}n_Q}{\mathrm{dt}} = k_{QF}n_T \quad (3)$$

1.Supplementary descriptions of exciton dynamics and kinetic equations (1)-(3).

For S_1 excitons formed through photo-excitation, they would directly decay via radiative (prompt and delayed fluorescence) or non-radiative (thermal dissipation) process, or convert into T_1 excitons via intersystem crossing (ISC) process, or join singlet-singlet annihilation (SSA) / singlet-triplet annihilation (STA) process. T_1 excitons formed through ISC from S_1 would directly decay via radiative (phosphorescence) or non-radiative (degradation or thermal dissipation) process, or convert into S_1 excitons via RISC process, or join STA/TTA process. Usually, SSA process consumes two S_1 exciton and produce one S_1 exciton, thus the coefficient of SSA process in equation (1) is 2-1=1 and STA process consumes one S_1 exciton, one T_1 exciton and produce one T_1 exciton, thus the coefficient of STA process in equation (2) is 0 while in equation (1) is 1, and as for TTA process, it would consume two triplet exciton and produce γ S_1 exciton and (1-γ) T_1 exciton. For all the materials studied in our work with $E_{S1}<2E_{T1}$, and $E_{T2}<2E_{T1}$, γ is 0.25. Of note, as the degradation tests were conducted under continuous UV illumination, the existence of polaron could be ignored. And just as the discussion in the introduction of main text, due to the final emission of TADF emitters is from singlet states not triplet states, in the simulation of TADF materials one could not mainly consider the decay process of triplet excitons like that in PH materials, but need full consideration of the complex decay process of singlet excitons as well, which brings more challenge to the simulations.

2. Supplementary discussions for the parameter choices in the numerical simulation.

In the parameters of simulation, since all the materials are blue emitters with high Photoluminescence Quantum Yield (PLQY), according to the energy gap law and PLQY=$k_{r,S}/(k_{r,S} + k_{nr,S} + k_{ISC})$, the $k_{nr,S}$ could be ignored, and due to all the TADF emitters do not show room temperature phosphoresce (RTP), $k_{r,T}$ is also neglectable. Values of $k_{r,S}$, k_{ISC}, k_{RISC}, and $k_{nr,T}$ were obtained from experiments based on the method reported by Adachi et al.[5]. According to the literatures, k_{SSA}, k_{STA}, and k_{TTA} were set as 1×10^{-12} cm^3

s^{-1}, 1×10^{-12} cm^3 s^{-1}, and 1×10^{-14} cm^3 s^{-1}, respectively and remained constant in simulation, the rationality of this treatment would be verified later. k_{QS} and k_{QT} were set as 1×10^{-9} cm^3 s^{-1} and 1×10^{-11} cm^3 s^{-1} at first according to the related literatures and finely tuned during numerical simulation.

3.Supplementary description for the simulation.

First, we typed the equation (1)-(3) into Matlab, and then input aforementioned each rate constant. k_{QF} was set as 1×10^{-3} s^{-1} at first. Since in the photo-degradation test, the rate of exciton decay is much larger than that of quencher formation, an equilibrium would establish among these photophysical process. Thus, we could get the $n_S(t)$, $n_T(t)$, and $n_Q(t)$ at any time t according to the iteration through Matlab. The output simulation results are like data in Supplementary Table 4. According to the comparison between simulation result and experimental results, we optimized the value of k_{QF} and made tiny adjustment to k_{QS} and k_{QT} to obtain well-fitted results (Fig. 4a). By this way, we could get the value of k_{QF}, k_{QS}, and k_{QT}.

Supplementary Table 5. The output simulation results of DMAC-DPS neat film

Time (s)	$n_Q(t)$ (cm^{-3})	$n_S(t)$ (cm^{-3})	$n_T(t)$ (cm^{-3})
…	…	…	…
8.51×10^{2}	1.55×10^{15}	5.79×10^{12}	1.73×10^{14}
1.03×10^{3}	1.85×10^{15}	5.61×10^{12}	1.67×10^{14}
1.33×10^{3}	2.32×10^{15}	5.35×10^{12}	1.59×10^{14}
1.62×10^{3}	2.76×10^{15}	5.12×10^{12}	1.52×10^{14}
…	…	…	…

4.**Supplementary discussions for the rationality of assumptions in numerical simulation.**

4.1. The assumption that the excitation intensity *I* is nearly constant during experiment. We compared the absorption spectrum of DMAC-DPS and 5CzBN before and after illumination, and found that their absorption intensity nearly unchanged (Supplementary Fig. 9). This result demonstrates the decay of PL was originated from quenching by quenchers other than direct vanishing of emitters. So, it is reasonable that the excitation intensity I is nearly constant during experiment.

4.2. The rationality of the simulated k_{QS} and k_{QT}. We measured the prompt lifetime (τ_p) of fresh and aged film of each material and compared the experimental τ_p with those calculated based on the simulation results according to equation (4) and (5). The simulation results were well fitted with the experiment results (Supplementary Fig. 10). As for the k_{QT}, values in our study are similar with those reported[20] by other researchers.

$$\tau_p(\text{fresh}) = \frac{1}{k_r + k_{ISC}} \tag{4}$$

$$\tau_p(\text{aged}) = \frac{1}{k_r + k_{ISC} + k_{QS} n_Q} \tag{5}$$

4.3 The verification of the deadly influence on material and devices degradation of very few quenchers formed by irreversible bond cleavage. According to the simulation results, the rate constant of chemical

reaction quencher formation by irreversible bond cleavage, k_{QF}~10^{-3} s^{-1} is 8~10 magnitude smaller than those of photophysical processes such as k_{RISC} ~10^5 s^{-1} and k_f ~10^7 s^{-1}. It seems that the chemical process of bond cleavage should have little effect on the materials and device degradation. Of note, it is widely accepted that the degradation of OLEDs originates from exciton quenching by quenchers rather than the direct vanishment of emitters, which was evidenced by the little change of absorption spectrum of DMAC-DPS and 5CzBN before and after degradation. Based on such a small k_{QF}, we got k_{QT}~10^{-11} cm^3 s^{-1}, k_{QS}~10^{-9} cm^3 s^{-1}, and n_Q~10^{15} cm^{-3}. In the circumstances, the apparent rate constant of T_1 excitons quenching, $k_{QT}n_Q$ is ~10^4 s^{-1}, which is comparable with other rate constant of T_1 excitons consumption, such as k_{RISC} (~10^5 s^{-1}). Likewise, for S_1 excitons, the obtained $k_{QS}n_Q$~10^6 s^{-1} is comparable with k_f (~10^7 s^{-1}) etc. Thus, the very few quenchers formed by irreversible bond cleavage indeed could have deadly influence on degradation of material and devices.

5.Supplementary discussions for the comparison between single exciton model and hot exciton models.

Take TTA as an example, in the hot exciton model (TTA), equation (3) of quencher formation changes to $\mathrm{d}n_Q/\mathrm{d}t = k_{QF}{}' n_T^2$. Compared with the equation (3) of $\mathrm{d}n_Q/\mathrm{d}t = k_{QF}n_T$, we could find that the rate of quencher formation is more sensitive to the change of triplet density (n_T^2 vs n_T), which would lead to the large degradation rate at the beginning part of degradation (~ tens of minutes). And as the accumulation of quenchers, the degradation rate of hot exciton model would slow down quickly at the ending part (4~5 h) since the larger quencher formation rate. Thus, if we let the degradation rate in hot exciton model close to that in single exciton model at the beginning part, it would be smaller at the ending part, and vice versa, if we let the degradation rate in hot exciton model close to that in single exciton model at the ending part, it would be larger at the beginning part. Indeed, as shown in Fig. 4c, compared with the single exciton model, no matter how we adjusted the parameters in hot exciton model, the model could only fit well either at the beginning part (hot exciton model 1) or the ending part (hot exciton model 1) of the experimental results, which further supports that TTA process is not the main quencher formation way. When the hot excitons were formed via SSA or STA, the equation (3) would change to $\mathrm{d}n_Q/\mathrm{d}t = k_{QF}n_S^2$ or $\mathrm{d}n_Q/\mathrm{d}t = k_{QF}n_S n_T$. The degradation rate in hot exciton models (SSA or STA) would also much larger than that in single exciton model at the initial part and would also be more sensitive to the density of excitons. Thus, the similar degradation behaviors and simulation results of SSA and STA models with TTA model could be expected.

Of note, we emphasized again that the neglect of SSA, STA, and TTA process is based on our experiment condition that the illumination intensity is ~2 mW cm^{-2} and the density of singlet and triplet excitons is 10^{13} ~ 10^{14} cm^{-3}, when the illumination intensity increases to ~ 10^3 mW cm^{-2} and the density of excitons increases to 10^{16} ~ 10^{17} cm^{-3}, the effect of SSA, STA, and TTA could no longer be ignored.

Supplementary References

1. Tsuchiya, Y. *et al.* Exact Solution of Kinetic Analysis for Thermally Activated Delayed Fluorescence Materials. *J. Phys. Chem. A* **125**, 8074–8089 (2021).
2. Lee, H. J., Lee, H. L., Han, S. H. & Lee, J. Y. Novel secondary acceptor based molecular design for superb lifetime in thermally activated delayed fluorescent organic light-emitting diodes through high bond energy and fast up-conversion. *Chem. Eng. J.* **427**, 130988 (2022).
3. Su, L. *et al.* High Fluorescence Rate of Thermally Activated Delayed Fluorescence Emitters for Efficient and Stable Blue OLEDs. *ACS Appl. Mater. Inter.* **12**, 31706–31715 (2020).
4. Song, W., Lee, I. & Lee, J. Y. Host Engineering for High Quantum Efficiency Blue and White Fluorescent Organic Light-Emitting Diodes. *Adv. Mater.* **27**, 4358–4363 (2015).
5. Zhang, Q. *et al.* Efficient blue organic light-emitting diodes employing thermally activated delayed fluorescence. *Nat. Photon.* **8**, 326–332 (2014).
6. Lin, T.-A. *et al.* Sky-Blue Organic Light Emitting Diode with 37% External Quantum Efficiency Using Thermally Activated Delayed Fluorescence from Spiroacridine-Triazine Hybrid. *Adv. Mater.* **28**, 6976–6983 (2016).
7. Liu, T. *et al.* Zero–Zero Energy-Dominated Degradation in Blue Organic Light-Emitting Diodes Employing Thermally Activated Delayed Fluorescence. *ACS Appl. Mater. Inter.* **14**, 22332–22340 (2022).
8. Ahn, D. H. *et al.* Rigid Oxygen-Bridged Boron-Based Blue Thermally Activated Delayed Fluorescence Emitter for Organic Light-Emitting Diode: Approach towards Satisfying High Efficiency and Long Lifetime Together. *Adv. Opt. Mater.* **8**, 2000102 (2020).
9. Cui, L.-S. *et al.* Controlling Synergistic Oxidation Processes for Efficient and Stable Blue Thermally Activated Delayed Fluorescence Devices. *Adv. Mater.* **28**, 7620–7625 (2016).
10. Ahn, D. H. *et al.* Highly efficient blue thermally activated delayed fluorescence emitters based on symmetrical and rigid oxygen-bridged boron acceptors. *Nat. Photon.* **13**, 540–546 (2019).
11. Zhang, D. *et al.* Efficient and Stable Deep-Blue Fluorescent Organic Light-Emitting Diodes Employing a Sensitizer with Fast Triplet Upconversion. *Adv. Mater.* **32**, 1908355 (2020).
12. Kondo, Y. *et al.* Narrowband deep-blue organic light-emitting diode featuring an organoboron-based emitter. *Nat. Photon.* **13**, 678–682 (2019).
13. Lee, D. R., Choi, J. M., Lee, C. W. & Lee, J. Y. Ideal Molecular Design of Blue Thermally Activated Delayed Fluorescent Emitter for High Efficiency, Small Singlet–Triplet Energy Splitting, Low Efficiency Roll-Off, and Long Lifetime. *ACS Appl. Mater. Inter.* **8**, 23190–23196 (2016).
14. Jeon, S. O. *et al.* High-efficiency, long-lifetime deep-blue organic light-emitting diodes. *Nat. Photon.* **15**, 208–215 (2021).
15. Cheng, C. *et al.* Efficient and stable deep blue thermally activated delayed fluorescent molecules based on a bipyridine acceptor core. *J. Mater. Chem. C* **9**, 3088–3095 (2021).
16. Kang, H. *et al.* Designing Stable Deep-Blue Thermally Activated Delayed Fluorescence Emitters through Controlling the Intrinsic Stability of Triplet Excitons. *Adv. Opt. Mater.* **10**, 2102309 (2022).
17. Cui, L.-S. *et al.* Fast spin-flip enables efficient and stable organic electroluminescence from charge-transfer states. *Nat. Photon.* **14**, 636–642 (2020).
18. Kim, M., Jeon, S. K., Hwang, S.-H. & Lee, J. Y. Stable Blue Thermally Activated Delayed Fluorescent Organic Light-Emitting Diodes with Three Times Longer Lifetime than Phosphorescent Organic Light-Emitting Diodes. *Adv. Mater.* **27**, 2515–2520 (2015).
19. Zhang, D., Cai, M., Zhang, Y., Zhang, D. & Duan, L. Sterically shielded blue thermally activated delayed fluorescence emitters with improved efficiency and stability. *Mater. Horizons* **3**, 145–151 (2016).

20. Lee, J. *et al.* Hot excited state management for long-lived blue phosphorescent organic light-emitting diodes. *Nat. Commun.* **8**, 15566 (2017).
